# A high-resolution 2D *J*-resolved NMR detection technique for metabolite analyses of biological samples

**DOI:** 10.1038/srep08390

**Published:** 2015-02-11

**Authors:** Yuqing Huang, Zhiyong Zhang, Hao Chen, Jianghua Feng, Shuhui Cai, Zhong Chen

**Affiliations:** 1 Department of Electronic Science, Fujian Provincial Key Laboratory of Plasma and Magnetic Resonance, State Key Laboratory of Physical Chemistry of Solid Surfaces, Xiamen University, Xiamen, Fujian 361005, China

## Abstract

NMR spectroscopy is a commonly used technique for metabolite analyses. Due to the observed macroscopic magnetic susceptibility in biological tissues, current NMR acquisitions in measurements of biological tissues are generally performed on tissue extracts using liquid NMR or on tissues using magic-angle spinning techniques. In this study, we propose an NMR method to achieve high-resolution *J*-resolved information for metabolite analyses directly from intact biological samples. A dramatic improvement in spectral resolution is evident in our contrastive demonstrations on a sample of pig brain tissue. Metabolite analyses for a postmortem fish from fresh to decayed statuses are presented to further reveal the capability of the proposed method. This method is a previously-unreported high-resolution 2D *J*-resolved spectroscopy for biological applications without specialised hardware requirements or complicated sample pretreatments. It provides a significant contribution to metabolite analyses of biological samples, and may be potentially applicable to *in vivo* samples. Furthermore, this method also can be applied to measurements of semisolid and viscous samples.

NMR spectroscopy has been proven as a powerful tool for metabolite analyses of biological samples^1^,^2^,^3^,^4^. Benefiting from the high-throughput information relevant to biochemical and biological processes and from the intrinsic noninvasiveness involved in its measurements, NMR spectroscopy has been successfully applied in various fields, including functional toxicology^5^,^6^, environmental science^7^,^8^, and nutrition studies^9^. For example, proton ( ^1^H) NMR spectra of biological fluids (such as blood, plasma or urine) are rich in metabolic information and thus are useful for studying endogenous metabolic changes caused by drug toxicities or diseases^10^,^11^. To date, 1D ^1^H NMR spectroscopy is one of the most popular techniques for metabolite studies. Advantages of 1D ^1^H NMR approaches include (a) relatively rapid spectral acquisition and (b) direct measurement of metabolite concentrations using a single internal standard^12^. However, due to the limited range of proton chemical shifts and a large number of resonances from various metabolites, spectral congestion, even severe overlapping of spectral peaks, is generally encountered in 1D NMR spectra of tissue extracts^13^ and biological fluids^14^. This limitation poses a considerable challenge for unique identification and quantification of metabolites.

Moving from 1D to 2D ^1^H NMR spectroscopy is a natural solution for spectral congestion because 2D ^1^H NMR provides more molecular information and more accurate metabolite specificities. However, the applications of most 2D NMR methods are generally limited by their longer acquisition time. The 2D *J*-resolved (*J*RES) ^1^H NMR spectroscopy^15^ can yield a 2D spectrum with efficient acquisitions for complex metabolite mixtures. This approach separates chemical shifts and *J*-couplings into two different spectral dimensions. A proton-decoupled 1D spectrum can be obtained from the skyline projection along the chemical shift dimension, facilitating metabolite assignments and quantifications. The provision of *J*-coupling information along the *J*-coupling dimension aids metabolite identifications because *J*-couplings are insensitive to physiological factors (such as temperature or pH value) *versus* chemical shifts^16^. Due to these advantages, 2D *J*RES spectroscopy has been broadly applied in metabolite analyses, such as studies examining urine^17^, cerebral spinal fluid^18^, and blood plasma^19^.

In addition to the aforementioned spectral acquisition strategy, spectral resolution is another significant factor because high-resolution NMR information is a prerequisite for the establishment of metabolite analyses. Metabolite studies of biological samples are generally performed on tissue extractions, aiming for high-resolution spectral information. However, tissue extractions only indirectly reveal metabolite information from original tissues and may not contain all metabolites due to complicated sample pretreatments^20^. If biological tissues are measured directly, the existence of macroscopic magnetic susceptibility will lead to magnetic field inhomogeneities. These inhomogeneities result in spectral line broadening, which conceals the spectral information necessary for metabolite analyses. The magic-angle spinning (MAS) technique^21^,^22^ provides an effective method for eliminating variations of macroscopic magnetic susceptibility in biological tissues. The MAS NMR spectra of biological tissues exhibit a spectral resolution similar to those of solution-state NMR spectra. However, specialised hardware is required for MAS experiments. Moreover, organic textures of biological tissues are generally destroyed by the fast spinning, which presents a challenge for studying intact biological tissues. In view of the resolution challenge in metabolite analyses of biological samples, there has been increasing demand for high-resolution methods that can be directly applied to intact biological samples, and that can be readily adapted to standard NMR spectrometer hardware.

In the present study, we present an NMR method, tentatively named DDF*J*RES, for the direct acquisition of high-resolution 2D *J*RES spectra from intact biological samples. This approach does not require complicated tissue pretreatments or specialised MAS equipment. The DDF*J*RES pulse sequence is designed based on the distant dipolar field (DDF) effect. It has been demonstrated that the DDF, which originates from magnetic dipole interactions within a correlation distance (typically in the range of 5 ~ 500 μm) in a sample^23^,^24^, can be utilised to improve NMR spectral resolution^25^,^26^. The effect of field inhomogeneities due to macroscopic magnetic susceptibility in biological samples can be removed in the 2D *J*RES spectra obtained by the DDF*J*RES method. Moreover, the DDF*J*RES method holds the same acquisition time as the standard 2D *J*RES technique, and it can be readily implemented on standard NMR spectrometers. Experiments on an aqueous solution were performed to demonstrate the implementation of the DDF*J*RES method. A pig brain tissue, a whole fish (Siamese algae eater), and shishamo smelt eggs (Fig. S1 in the Supplementary Information) were used to examine the capability of the DDF*J*RES method. In addition, a semisolid sample of fruit jelly (Fig. S2 in the Supplementary Information) and a viscous sample of facial cream (Fig. S3 in the Supplementary Information) were used to further show the applicability of the DDF*J*RES method.

## Results

### Aqueous solution

**Figure 1:**
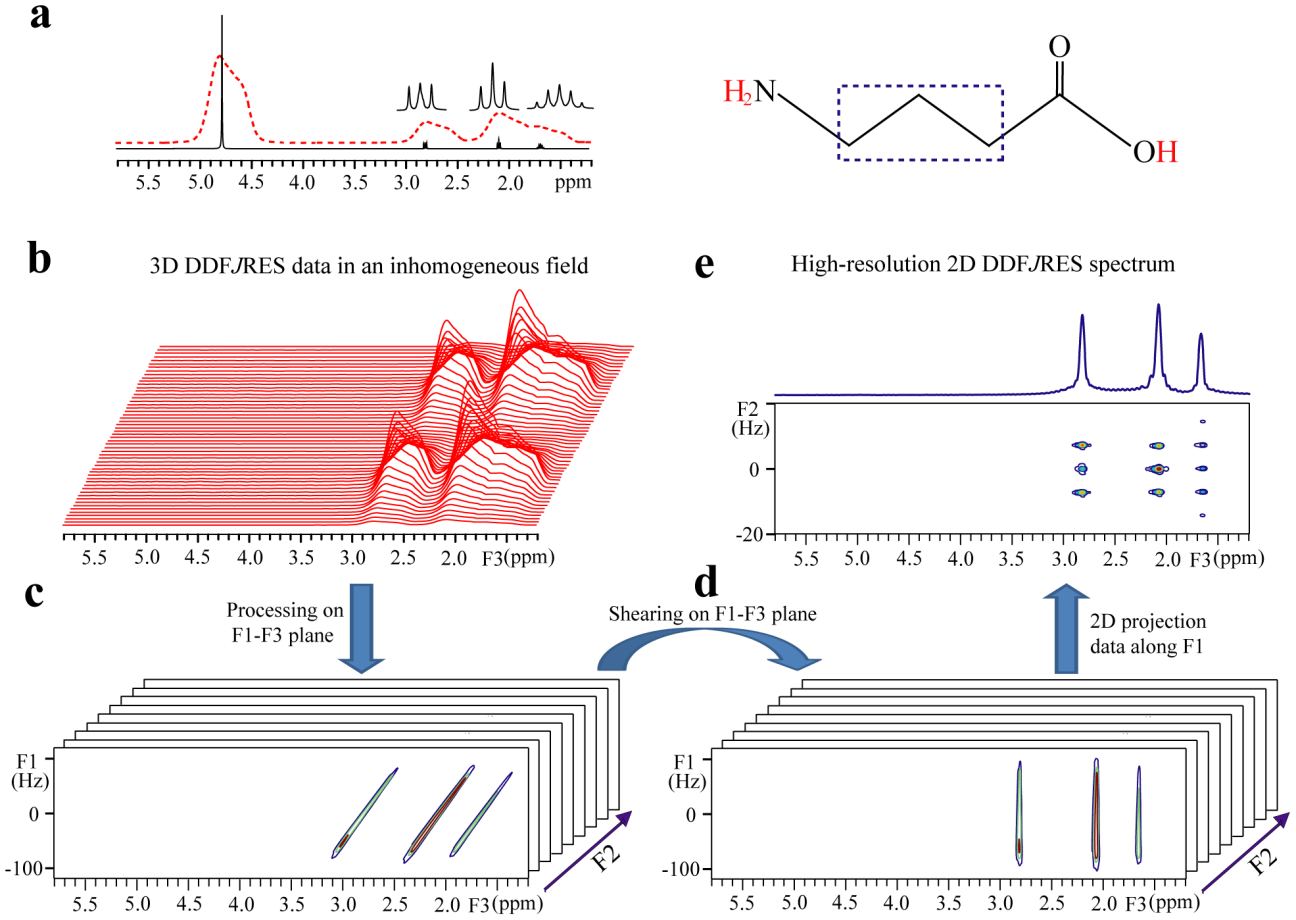
Illustration of the DDF*J*RES processing using the data from a GABA aqueous solution. (a) 1D proton spectrum with expanded multiplets in a well-shimmed homogeneous field (the black solid line) and in an inhomogeneous field where the FWHM of water resonance was deliberately degraded to 200 Hz (the red dotted line). (b) 3D DDF*J*RES data presented as stacked 1D spectra along the F3 frequency domain. (c) A batch of 2D spectra after data processing in F1 and F3 dimensions. (d) 2D spectra corresponding to (c) after a shearing process. (e) High-resolution 2D *J*RES spectrum constructed using F2 and F3 dimensions after 2D projection along the F1 dimension. The molecular structure of GABA is shown on the top right of the frame, together with observable protons marked by a dotted box.

Major steps for obtaining a high-resolution DDF*J*RES spectrum are illustrated using experimental data for a 0.8 M γ-aminobutyric acid (GABA) aqueous solution (Fig. 1). The molecular structure of GABA is given and three observable protons are marked by a dotted box. A 1D NMR spectrum with three expanded multiplets acquired in a well-shimmed magnetic field is shown as a black solid line, and the spectrum acquired in an inhomogeneous field is presented in a red dotted line (Fig. 1a). The inhomogeneous magnetic field is created by deliberately degrading the shimming currents and the full width at half maximum (FWHM) of the water peak at 4.80 ppm is 200 Hz. The DDF*J*RES experiment is carried out under this inhomogeneous field. After data acquisition, a batch of 1D spectra along the F3 frequency domain are obtained (Fig. 1b). The water peak is completely suppressed in these 1D spectra. Due to the field inhomogeneity, all 1D spectra suffer from line broadening, with 200 Hz FWHM for the peak at 2.83 ppm. Initially, the processing of the 3D DDF*J*RES data is performed on F1 and F3 dimensions, and *ni* 2D spectra are obtained (Fig. 1c). These 2D spectra are bound together by *J*-coupling evolution along the F2 dimension. Inhomogeneous line broadening occurs along F1 and F3 dimensions, resulting in signal streaks that are parallel to one another. A shearing process on the F1–F3 plane is then performed on all 2D spectra to eliminate the inhomogeneous line broadening along the F3 dimension (Fig. 1d). A projection along the F1 dimension for these 2D spectra is performed to retain the chemical shift information in the F3 dimension. In addition, the *J*-coupling information is presented in the F2 dimension after a 1D Fourier transformation. Consequently, a high-resolution 2D *J*RES spectrum, constructed from F2 and F3 dimensions, is obtained. A complete separation of chemical shifts and *J*-couplings is achieved by a rotation of 45° (Fig. 1e). It can be seen that the inhomogeneous line broadening is significantly reduced. The FWHM of the peak at 2.83 ppm is reduced from 200 Hz to 30 Hz along the F3 dimension and to 2.8 Hz along the F2 dimension.

### Pig brain tissue

**Figure 2:**
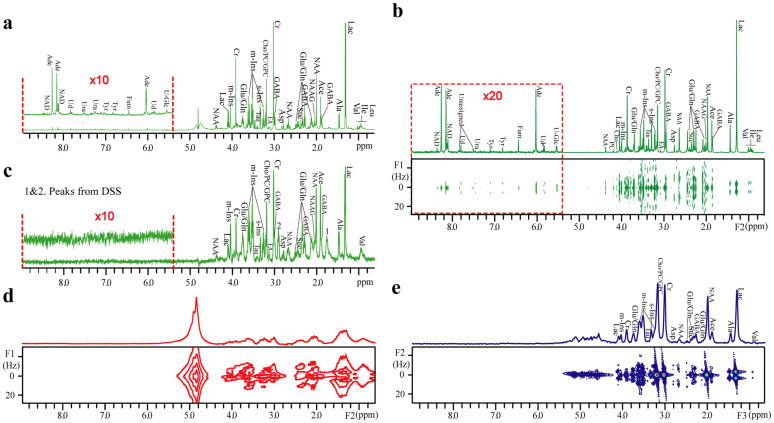
Contrastive NMR results on pig brain tissue. (a) 1D water-presaturated spectrum of the tissue extraction. (b) 2D water-presaturated *J*RES spectrum of the tissue extraction and its projection along the F2 axis. (c) 1D water-presaturated MAS spectrum of the tissue packed in a 4-mm ZrO_2_ rotor acquired using a Nano probe. (d) 2D water-presaturated *J*RES spectrum and its projection along the F2 axis; (e) 2D DDF*J*RES spectrum and its 1D *J*-decoupled projection along the F3 axis. The sample for (d) and (e) is a piece of intact tissue fitted in a 5-mm NMR tube. The spectral region from 5.40 to 9.00 ppm is expanded for (a), (b), and (c). Assigned metabolites are marked in (a), (b), (c), and (e), respectively.

A contrastive result obtained from the DDF*J*RES and the traditional NMR methods is executed on a sample of pig brain tissue (Fig. 2). The 1D spectrum (Fig. 2a) and the 2D *J*RES spectrum (Fig. 2b) acquired from a tissue extraction in a well-shimmed homogeneous field provides NMR information for metabolite analyses in a high-resolution manner. For the 1D spectrum of the tissue extraction (Fig. 2a), the FWHM of creatine (Cr) at 3.01 ppm shows 1.9 Hz in the phase-sensitive display mode. All resonances are well-resolved, and metabolites are explicitly assigned, even for weak metabolites in the low field spectral region (the expanded region from 5.40 ppm to 9.00 ppm). For the 2D *J*RES spectrum (Fig. 2b), chemical shifts and *J* couplings are separated along F1 and F2 dimensions, respectively, and a 1D *J*-decoupled projection along the F2 dimensions is presented. The FWHM of Cr at 3.01 ppm in the 1D *J*-decoupled projection is 3.9 Hz in the absolute-value display mode. Similarly, metabolites are assigned in the whole spectral region (0.65 ~ 9.00 ppm), and related *J*-coupling constants can be measured along the F1 dimension. With the aid of MAS techniques, a high-resolution 1D spectrum of the brain tissue is obtained (Fig. 2c). The FWHM of Cr at 3.02 ppm in 1D MAS spectrum is 5.3 Hz in the phase-sensitive display mode. Metabolites in the high field spectral region (0.65 ~ 5.00 ppm) are assigned. Compared to results of the tissue extraction (Fig. 2a, b), the 1D MAS spectrum of the tissue (Fig. 2c) loses some metabolites, such as weak metabolites in the low field spectral region (5.40 ppm ~ 9.00 ppm), isoleucine (Ile) at 0.92 ppm, and leucine (Leu) at 0.95 ppm.

The standard 2D *J*RES technique can be applied to the tissue extraction for high-resolution information. However, when it is directly applied to an intact brain tissue sample, the spectral resolution is severely degraded and the desired NMR information is lost (Fig. 2d). In the 2D *J*RES spectrum of the intact brain tissue, the FWHM of the water peak at 4.85 ppm along the F2 dimension is 100 Hz, resulting in the loss of spectral information for metabolite assignments. Although the inhomogeneous line broadening could be removed along the F1 dimension in the 2D *J*RES spectrum, the spectral peak overlap in the F2 dimension renders the measurement of accurate *J*-coupling information challenging. By contrast, a high-resolution 2D *J*RES spectrum is obtained from the same intact brain tissue using the DDF*J*RES method. After the same data processing (Fig. 1), a 2D DDF*J*RES spectrum, with its 1D *J*-decoupled projection along the F3 axis, is obtained (Fig. 2e). Compared with the standard 2D *J*RES spectrum (Fig. 2d), the spectral resolution is significantly enhanced in the 2D DDF*J*RES spectrum (Fig. 2e). The FWHM of Cr at 3.01 ppm in its 1D *J*-decoupled projection shows 30 Hz in the absolute-value display mode, proving satisfactory for metabolite analyses^27^. Similarly to the 1D MAS spectrum of the tissue, weak metabolites in the spectral region from 5.4 ppm to 9.0 ppm, isoleucine (Ile) at 0.92 ppm, and leucine (Leu) at 0.95 ppm are lost in the DDF*J*RES spectrum. In addition, resonances from GABA at 3.01 ppm and 1.89 ppm, ethanolamine (EA) at 3.13 ppm, N-acetyl aspartate (NAA) at 4.38 ppm, and N-acetyl aspartate glutamate (NAAG) at 2.05 ppm vanish in the DDF*J*RES spectrum due to the limited spectral resolution. Except for these metabolites, all metabolites assigned in the DDF*J*RES spectrum (Fig. 2e) are the same as those assigned in the spectra of the tissue extraction (Fig. 2a, b) and the 1D MAS spectrum of the tissue (Fig. 2c). In addition, the *J*-coupling information is distinctly presented in the F2 dimension of the DDF*J*RES spectrum. A comparison among 2D standard *J*RES spectrum of the tissue extraction (Fig. 2b), the 1D MAS spectrum of the tissue (Fig. 2c), and the 2D DDF*J*RES spectrum of a piece of intact tissue (Fig. 2e) is performed, and the ^1^H chemical shifts, multiplet patterns, and *J*-coupling constants of measured metabolites are presented in Table 1. Forty-nine peaks in the 2D *J*RES spectrum of the tissue extraction are assigned to 27 metabolites, 23 peaks in the 2D DDF*J*RES spectrum of a piece of intact tissue are assigned to 15 metabolites, and 28 peaks in the 1D MAS spectrum of the tissue are assigned to 17 metabolites. Clearly, the 2D *J*RES spectrum of the tissue extraction provides maximum metabolite information. All peaks observed in the 2D DDF*J*RES spectrum exhibit chemical shifts, *J*-coupling constants, and multiplet patterns similar to those in the 1D MAS spectrum.

### Fish

**Figure 3:**
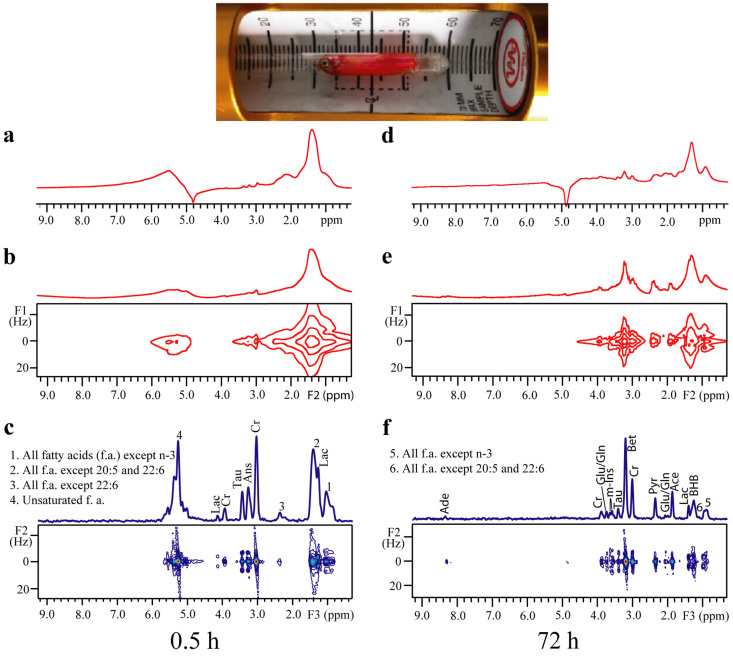
Postmortem study of a whole fish (Siamese algae eater) fitted in a 5-mm NMR tube. (a–c) 1D water-presaturated, standard 2D water-presaturated *J*RES, and 2D DDF*J*RES spectra, respectively at 0.5 h after the sample preparation. (d–f) Spectra corresponding to (a–c) at 72 h after the sample preparation. Metabolite assignments are shown in the 2D DDF*J*RES spectra. A photo of the sample is provided at the top of the frame.

Results of a whole postmortem Siamese algae eater using 1D water-presaturated sequence, standard 2D water-presaturated *J*RES sequence, and DDF*J*RES method are presented (Fig. 3). The whole fish is fitted into a 5-mm NMR tube, as shown in the top of the figure. In this study, measurements are performed at two different time-points, 0.5 h (fresh status) and 72 h (decayed status) after the sample preparation. Figure 3a, b, and c display 1D water-presaturated, standard 2D water-presaturated *J*RES, and 2D DDF*J*RES spectra of the fish at the fresh status, respectively, while Figure 3d, e, and f show the corresponding spectra at the decayed status. The field inhomogeneity of macroscopic magnetic susceptibility inside the fish body impacts 1D NMR spectral information (Fig. 3a, d). The FWHM of the peak at 1.40 ppm gives 120 Hz (Fig. 3a) and the FWHM of the peak at 1.35 ppm shows 100 Hz (Fig. 3d) in the phase-sensitive display mode. Although two main resonances from fatty acids (f.a.) at 1.40 ppm and 5.42 ppm, along with their changes during the two different statuses, are recognisable, it remains unlikely to obtain information for other metabolites. Even in 2D water-presaturated *J*RES spectra (Fig. 3b, e), spectral analyses encounter a similar predicament caused by the inhomogeneous line broadening.

The 2D DDF*J*RES spectra (Fig. 3c, f) reveal satisfactory *J*RES NMR information for the whole Siamese algae eater. The spectral resolution is enhanced, and the desired information regarding chemical shifts and *J*-couplings becomes available. The FWHM of small-molecule metabolites (e.g. Cr at 2.99 ppm) is reduced to 31 Hz, and the FWHM of f.a. is larger than this value because of the intrinsically shorter relaxation time in fatty tissues. Compared with results from the 1D water-presaturated sequence and the standard 2D water-presaturated *J*RES sequence, signal peaks from f.a. and small-molecule metabolites are well-resolved and could be assigned in the F3 dimension of 2D DDF*J*RES spectra. The *J*-coupling measurements can be achieved in the F2 dimension. In detail, 4 peaks of f.a. and 6 peaks of other small-molecule metabolites are given in the 2D DDF*J*RES spectrum at the fresh status (Fig. 3c), while 2 peaks of f.a. and 13 peaks of other small-molecule metabolites are obtained in that at the decayed status (Fig. 3f). A comparison of the metabolite information acquired from the DDF*J*RES and standard 2D *J*RES methods is presented in Table 2. These results convincingly suggest the superiority of the DDF*J*RES method over standard 2D *J*RES methods for real biological samples.

## Discussion

From aforementioned experimental results, evidently, the DDF*J*RES method can be used to recover high-resolution 2D *J*RES information from inhomogeneous fields, such as those caused by the observed macroscopic magnetic susceptibility in biological samples. Currently, tissue extraction and MAS techniques are used to retrieve high-resolution spectral information for metabolite analyses of biological samples. However, for some specific biological samples which cannot be invaded and should be kept intact during the NMR acquisition, tissue extraction and MAS techniques will be limited. Based on pulse sequence design and simple data processing, the DDF*J*RES method provides an effective method for high-resolution 2D *J*RES measurements on intact biological tissues. This method can be readily adapted to commonly available NMR spectrometers without any specialised hardware requirements. Our experiments are performed on a typical NMR spectrometer (500-MHz Varian NMR system with a 54-mm narrow bore), and objective samples should fit in a 5-mm NMR tube. Therefore, biological samples with appropriate sizes are selected for our measurements, such as pig brain tissue, small fish (Siamese algae eater), and shishamo smelt eggs (Supplementary Information). Due to low demands for specialised hardware, the DDF*J*RES method may be also applicable to other NMR or MRI systems equipped with broad inner bores, which allow for measurements on biological samples with fewer size limitations.

We use a GABA aqueous solution in a deliberately deshimmed inhomogeneous field to illustrate the implementation of the DDF*J*RES method. Experimental results verify that high-resolution 2D *J*RES spectra can be obtained using the DDF*J*RES method. Although the verification is based on a simple aqueous solution, it is intuitively applicable to complicated biological systems, in which metabolites are analogous to GABA and the field inhomogeneity originating from macroscopic magnetic susceptibility behaves similarly to the deliberately deshimmed field inhomogeneity. The signal intensity is a key factor for metabolite analyses in biological samples, and the signal-to-noise ratio (SNR) is generally used to estimate the performance of NMR acquisition methods in signal intensity. An SNR calculation was performed for the 2D DDF*J*RES spectrum of the aqueous solution (Fig. 1e). The SNR, measured using the intensity of the peak at 2.83 ppm divided by noises between 5.2 and 5.7 ppm in the 1D projection along the F3 dimension, is 24.3. In the same inhomogeneous field, a SNR value of 116.7 is calculated for the standard 2D *J*RES spectrum. Therefore, the SNR in the DDF*J*RES spectrum presents 20.8% of that in the standard 2D *J*RES spectrum. Signals from the DDF*J*RES method are lower than those from the standard *J*RES technique. This signal intensity loss is unavoidable because the DDF-based resolution enhancement in inhomogeneous fields is achieved at the cost of signal intensity^25^. Improvements in signal intensity may be achieved by using the dynamic nuclear polarisation technique^28^.

In general, it is important and necessary for metabolite analyses of biological samples to rapidly acquire NMR spectra. Although 3D acquisition is required for the DDF*J*RES method, the utilisation of spatial encoding and decoding techniques^29^ can supply the F1 dimension, which is sampled in a single scan. Thus the acquisition time of the DDF*J*RES method only depends on the range of *J* coupling splitting along the F2 dimension, and it is the same as the standard 2D *J*RES technique. In the previous study, an NMR method, named iDQC*J*RES, was also proposed for 2D *J*RES applications on biological tissues^30^. The iDQC*J*RES method is also based on the DDF effect and holds the similar signal intensity with the DDF*J*RES method. However, two normal indirect evolution periods, *t*_1_ and *t*_2_, are included and a standard 3D acquisition is required in the iDQC*J*RES experiment. Compared to the iDQC*J*RES method, the acquisition time of the DDF*J*RES method is significantly improved, particularly for samples with large field inhomogeneity, and more transients can be acquired in DDF*J*RES experiments. Due to the intrinsic resolution defect of the acquisition mode in the spatial encoding and decoding scheme^31^, the resolution remains low in the F3 dimension of the DDF*J*RES spectra. Based on the balance between the acquisition time and the spectral resolution, the DDF*J*RES method has been carefully designed for applications involving intact biological samples. The DDF*J*RES spectra of pig brain tissue (Fig. 2e), a whole fish (Fig. 3c, f), and shishamo smelt eggs (Fig. S1d in the Supplementary Information) demonstrate that resonances are well-resolved for metabolite analyses.

As observed for the pig brain tissue, all major metabolite information can be observed using the DDF*J*RES method (Fig. 2e and Table 1). Compared to results acquired from tissue extraction in a well-shimmed field (Fig. 2a, b), weak metabolite signals in low field region (5.40 ~ 9.00 ppm) are lost in both 1D MAS spectrum of the tissue (Fig. 2c) and 2D DDF*J*RES spectrum of a piece of intact tissue (Fig. 2e). In addition, five peaks in high field region (0.65 ~ 5.00 ppm) remain unobservable in the 2D DDF*J*RES spectrum. Although results obtained from the DDF*J*RES method cannot match those acquired from tissue extraction in a well-shimmed field, it can be performed on the intact tissue, without causing invasive damages to internal structures of the tissue. The spectral quality is significantly improved in the 2D DDF*J*RES spectrum compared to the standard 2D *J*RES spectrum acquired from the same intact tissue. Most metabolites assigned in the 2D DDF*J*RES spectrum are the same as those in the 1D MAS spectrum. Hence, the DDF*J*RES method presents an alternative to tissue extraction and MAS techniques for cases in which objective biological samples should be kept intact during the whole NMR acquisition.

The pig brain tissue used in our experiments belongs to an excised biological sample and is quite different from real biological samples with integrated organismal parts. Therefore, a postmortem study of a whole Siamese algae eater is performed. In this study, the information (Fig. 3c, f and Table 2) provided by the DDF*J*RES method can be used for metabolite analyses of fish tissues during the postmortem period. Intensities of f.a. signals dramatically decrease from the fresh status to postmortem status (Fig. 3c to 3f). Particularly, signals of unsaturated f.a. at 5.24 ppm and all f.a. except 22:6 at 2.39 ppm vanish in the spectrum obtained at 72 h (Fig. 3f). This observation accords with previous measurements of lipid oxidations^32^. The observation of metabolites that are present only in Fig. 3f implies complex biochemical processes during the postmortem period. Some metabolites, such as f.a., may be potential biomarkers for the decay process and quantitative determinations of these metabolite variations could be used for freshness evaluations. Freshness is an important factor for the quality of fish or fishery products. A large number of methods have been used for fish freshness evaluations during postmortem storage, such as sensory evaluation and physical measurements^33^. These methods are associated with both advantages and disadvantages in practical applications and are complementary for comprehensive freshness assessments. The DDF*J*RES method, as a noninvasive and convenient method, may provide a competitive tool for freshness evaluation of fishery products with rapid nutritional component analyses and intrinsic biomarkers.

To further exemplify the applicability of the DDF*J*RES method, we perform experiments on a sample of intact shishamo smelt eggs. This sample is obtained from a local caviar factory. An invasive and convenient method for measurements of main metabolites in the shishamo smelt eggs is beneficial to the qulitiy control of caviar product. In the DDF*J*RES experiment, the sample is directly fitted into a 5-mm NMR tube and then measured on a 500-MHz Varian NMR system, without invasive damages. Results (Supplementary Information) further verify the capacity of DDF*J*RES in recovering high-resolution spectral information on intact biological samples.

To show the feasibility of the DDF*J*RES method in other application fields besides metabolite analyses of biological samples, we also extend the DDF*J*RES method to the semisolid and viscous samples. Semisolid and viscous samples, such as food materials, cosmetic materials, or organic chemical materials, are generally characterized by the restricted molecular mobility, thus inhomogeneous line broadening exists and well-resolved spectral information is hardly obtained by using the traditional NMR methods. A semisolid sample of fruit jelly and a viscous sample of facial cream are chosen for the DDF*J*RES measurements. Both experimental results of the fruit jelly (Fig. S2 in the Supplementary Information) and those of the facial cream (Fig. S3 in the Supplementary Information) suggests that high-resolution spectral information for chemical component analyses of the objective samples can be obtained by using the DDF*J*RES method.

In conclusion, a previously-unreported NMR method named DDF*J*RES is proposed to obtain high-resolution 2D *J*RES information for metabolite analyses directly from intact biological samples. Compared to the traditional NMR methods of tissue extraction and MAS, no specialised hardware requirements or complicated sample pretreatments are required for the DDF*J*RES experiments. Furthermore, the DDF*J*RES method holds the same acquisition time as standard 2D *J*RES technique. The capability of the DDF*J*RES method is demonstrated by experiments of three biological samples, a semisolid sample (Supplementary Information), and a viscous sample (Supplementary Information). This method is readily implemented on standard NMR spectrometers, and is potentially applicable to biological samples or other semisolid and viscous samples on NMR or MRI system equipped with broad inner bores.

## Methods

### DDF*J*RES pulse sequence

**Figure 4:**
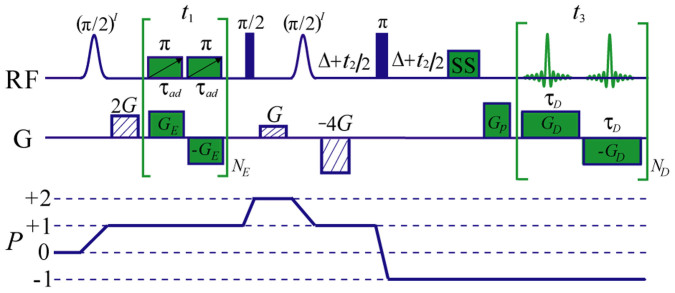
Pulse sequence diagram of DDF*J*RES. Gauss-shaped pulses represent solvent-selective RF pulses, full vertical bars indicate non-selective RF pulses, dashed rectangles represent coherence selection gradients, and “SS” indicates the solvent suppression module. The first evolution period *t*_1_ consists of the spatially encoded module (bipolar encoding gradients *G_E_* and relevant adiabatic frequency-swept π pulses), and the acquisition period *t*_3_ consists of the spatially decoded module (bipolar decoding gradients *G_D_*). The second evolution period *t*_2_ is a conventional spin echo scheme. *G_p_* is a pre-acquisition purge gradient. The desired coherence transfer pathway is also given.

A diagram of the DDF*J*RES pulse sequence is presented in Fig. 4. Three coherence selection gradients (CSGs) with an area ratio of 2:1:−4 are applied along the z-direction to select the desired coherence transfer pathway. The pulse sequence starts with a solvent-selective (π/2)*^I^* pulse. Subsequently, a time-constant spatial encoding module^29^, consisting of bipolar encoding gradients (+*G_E_*, −*G_E_*) and relevant adiabatic frequency-swept π pulses, serves as the first evolution period *t*_1_ and achieved *t*_1_ increments sampled in a single scan. The second non-selective π/2 pulse excites solute and solvent magnetisations, achieving coherence transfer from +1 to +2. A solvent-selective (π/2)*^I^* pulse rotates the solvent magnetisation onto the z-axis to produce the required DDF. Similar to the standard 2D *J*RES method, the second evolution period *t*_2_ is equally divided by a non-selective π pulse to form a spin echo scheme, retaining *J*-coupling for signals. Finally, in the acquisition period *t*_3_, observable signals after the DDF effect are acquired using spatial decoding modules in an echo planar imaging (EPI) manner^29^, consisting of a series of bipolar decoding gradients (+*G_D_*, −*G_D_*). A pre-acquisition purge gradient *G_p_* is utilised to adjust the echo locations. A excitation sculpting scheme is employed to achieve solvent suppression (SS)^34^ for biological samples. The theoretical expression for signals from the DDF*J*RES sequence was derived (see section I in the Supplementary Information for more details).

The method and experiments were carried out in accordance with the approved guidelines. All experimental protocols were approved by the Institutional Review Board at Xiamen University, Xiamen, China.

### NMR hardware

All experiments were performed on a 500-MHz Varian NMR spectrometer (Varian, Palo Alto, California, USA) equipped with a 5-mm ^1^H XYZ indirect detection probe with 3D gradient coils and a GHX Nano probe with a z-gradient coil.

### Aqueous solution

A 0.8 M γ-Aminobutyric acid (GABA, C_4_H_9_NO_2_) aqueous solution was used to demonstrate implementation details of the DDF*J*RES method. The homogeneity of magnetic field was deliberately degraded by altering Z1 shimming coils current. The FWHM of the broad water peak at 4.80 ppm was 200 Hz. The DD*J*RES sequence was applied in this inhomogeneous field. The width of the π/2 hard RF pulse was 10.2 μs, the solvent-selective π/2 pulse was a Gaussian shape with a width of 8.1 ms, and parameters (strength × duration) of the CSGs were *G* × *δ* = 0.1 T/m × 1.2 ms. Parameters for the crusher gradients in the SS module were *G*_1_ × δ′ = 0.07 T/m × 1.0 ms and *G*_2_ × δ′ = 0.24 T/m × 1.0 ms. Parameters for the spatial encoding and decoding gradients were *G_E_* × *τ_ad_* = 0.058 T/m × 10 ms and *G_D_* × *τ_D_* = 0.08 T/m × 0.217 ms, resulting in spectral widths of 250 Hz in the F1 dimension (the evolution period *t*_1_ sampled in a single scan) and 2300 Hz in the F3 dimension (the acquisition period *t*_3_). A spectral width of 40 Hz was used in the F2 dimension (the conventional evolution period *t*_2_) with 20 increments. A 2-s relaxation delay and 2 transients were applied, with a total acquisition time of 1.67 min. The 3D DDF*J*RES data were zero-filled to 128 × 64 × 2048 matrix in F1, F2, and F3 dimensions, respectively prior to data processing. A Gaussian window function was applied on both F1 and F3 dimensions.

### Pig brain tissue

To test the applicability of the DDF*J*RES method for biological tissues with intrinsic macroscopic magnetic susceptibility, we performed measurements on a sample of pig brain tissue. Three types of specimens were prepared from the pig brain tissue: tissue extraction using a water-methanol-chloroform system^35^ and taking the hydrophilic extract of low molecular weight metabolites^36^, a tiny bit of tissue (~15 mg) packed into a 4-mm ZrO_2_ rotor with a cylindrical insert, and a piece of intact tissue (~250 mg) fitted into a 5-mm NMR tube. For the tissue extraction, the standard 1D water-presaturated sequence and the standard water-presaturated 2D *J*RES sequence were used and experiments were performed in a well-shimmed magnetic field. The 1D water-presaturated spectrum was acquired with a 4500-Hz spectral width, a 2.5-s relaxation delay, a 10-μs π/2 pulse length, and 64 transients in 2.67 min. The standard 2D water-presaturated *J*RES spectrum was acquired with 30 × 3590 points for spectral widths of 50 Hz × 4500 Hz (F1 × F2), a 2.5-s relaxation delay, a 10-μs π/2 pulse length, 64 transients, and a total acquisition time of 80 min. The tissue packed into a 4-mm ZrO_2_ rotor was used for the MAS NMR experiment using a GHX Nano probe with a z-gradient. The spin rate of the rotor was regulated at 2,000 ± 10 Hz. Similarly, the standard 1D water-presaturated sequence was used to record a 1D MAS ^1^H NMR spectrum, using the same parameters employed in the tissue extraction experiment. For the piece of intact tissue fitted in a 5-mm NMR tube, the standard 2D water-presaturated *J*RES sequence and the DDF*J*RES sequence were applied. Experiments were performed without any field shimming. The standard 2D water-presaturated *J*RES spectrum was acquired with 30 × 640 points for spectral widths of 50 Hz × 4000 Hz (F1 × F2), a 2.5-s relaxation delay, a 10-μs π/2 pulse length, 8 transients, and a total acquisition time of 10 min. In the DDF*J*RES experiment, pulse parameters were an 11-μs π/2 hard pulse length and a 6.0-ms π/2 Gaussian pulse length, and parameters for the CSGs and the SS module were the same as those used in the aqueous solution measurement. Parameters for the spatial encoding and decoding modules were *G_E_* × *τ_ad_* = 0.022 T/m × 10 ms and *G_D_* × *τ_D_* = 0.051 T/m × 0.125 ms, resulting in spectral widths of 120 Hz and 4000 Hz in the F1 and F3 dimensions. A total of 30 increments were acquired for a 50-Hz spectral width in the F2 dimension, with a 2.5-s relaxation delay and 8 transients; the total acquisition time was 10 min.

### Fish

To demonstrate the feasibility of DDF*J*RES on real biological samples, we performed a postmortem study on a whole fish (Siamese algae eater) fitted into a 5-mm NMR tube. Both DDF*J*RES and standard water-presaturated 2D *J*RES sequences were used to investigate metabolic changes in the fish body at two different time-points: 0.5 h (fresh status) and 72 h (decayed status) after the sample preparation. For the standard water-presaturated 2D *J*RES experiment, 30 × 500 points were sampled with spectral widths of 50 Hz × 5000 Hz (F1 × F2), a 2.5-s relaxation delay, a 10-μs π/2 pulse length, and 16 transients, resulting in a total acquisition time of 20 min. In the DDF*J*RES experiment, pulse parameters were a 12-μs π/2 hard pulse length and a 6.5-ms π/2 Gaussian pulse length, and parameters for the CSGs and the SS module were the same as those used in the aqueous solution experiment. Parameters for the spatial encoding and decoding modules were *G_E_* × *τ_ad_* = 0.027 T/m × 10 ms and *G_D_* × *τ_D_* = 0.052 T/m × 0.1 ms, resulting in spectral widths of 150 Hz and 5000 Hz in F1 and F3 dimensions. A total of 30 increments were acquired for a 50-Hz spectral width in the F2 dimension, with a 2.5-s relaxation delay, 16 transients, and a total acquisition time of 20 min.

### Data processing

All DDF*J*RES data were processed using a custom-written program on MATLAB 7.8 (see section II in the Supplementary Information for detailed mathematical description on data processing).

## Author Contributions

Y.H. involved all the processes of designing the NMR pulse sequence, performing experiments, interpreting data, and writing the manuscript. Z.Z. involved the processes of designing the NMR pulse sequence and interpreting data. H.C. involved the process of performing experiments. J.F. and S.C. involved the process of writing the manuscript. Z.C. involved the processes of designing the NMR pulse sequence and writing the manuscript. All authors reviewed the manuscript.

## Supplementary Material

Supplementary Information Supplementary information

## Figures and Tables

**Table 1:** Assignments of 2D *J*RES spectrum from the pig brain tissue extraction, 2D DDF*J*RES spectrum from a piece of intact pig brain tissue, and 1D MAS spectrum from the pig brain tissue

		*J*RES/DDF*J*RES/MAS*1		
Metabolites	Groups	^1^H chemical shifts (ppm)	Multiplet patterns*2	*J*-couplings (Hz)
Isoleucine (Ile)	-CH_3_	0.92/n.o./n.o.*3	t/n.o./n.o.	7.4/n.o./n.o.
	-CH_3_	0.99/n.o./n.o.	d/n.o./n.o.	7.1/n.o./n.o.
Leucine (Leu)	-CH_3_	0.95/n.o./n.o.	t/n.o./n.o.	6.0/n.o./n.o.
Valine (Val)	-CH_3_	0.94/0.94/0.93	d/d/d	7.2/7.1/7.1
	-CH_3_	1.02/n.o./n.o.	d/n.o./n.o.	7.2/n.o./n.o.
Lactate (Lac)	-CH-C**H**_3_	1.31/1.31/1.31	d/d/d	7.1/7.1/7.2
	-C**H**-CH_3_	4.09/4.09/4.10	q/q/q	7.1/7.1/n.o.
Alanine (Ala)	-CH_3_	1.46/1.48/1.47	d/d/d	7.2/7.3/7.2
Acetate (Ace)	-CH_3_	1.90/1.91/1.90	s/s/s	-/-/-
N-Acetyl aspartate (NAA)	-CH_3_	2.00/2.01/2.00	s/s/s	-/-/-
	-CH-C**H**_2_	2.67/2.69/2.68	dd/dd/dd	4.1,15.6/3.9,15.6/4.1,15.8
	-C**H**-CH_2_	4.38/n.o./4.38	m/n.o./m	4.0/n.o./3.8
NAAG*4	-CH_3_	2.04/n.o./2.05	s/n.o./s	-/n.o./-
γ-Aminobutyric acid (GABA)	-CH_2_-C**H**_2_	1.88/n.o./1.89	m/n.o./n.o.	7.4/n.o./n.o.
	-C**H**_2_-CH_2_	2.28/2.29/2.29	t/t/t	7.4/7.4/7.3
	-CH_2_	3.00/n.o./3.01/	t/n.o./t.	7.1/n.o./7.0
Glutamate/Glutamine (Glu/Gln)	-CH_2_	2.05/n.o./n.o.	m/n.o./n.o.	7.4/n.o./n.o.
	-CH_2_	2.08/n.o./n.o.	m/n.o./n.o.	7.3,12.4/n.o./n.o.
	-CH_2_	2.11/2.11/2.11	m/m/m	7.6/7.8/7.4
	-CH_2_	2.33/2.34/2.33	m/m/m	3.5,7.1/3.4,7.3/7.6
	-CH_2_	2.48/2.49/2.48	m/m/m	7.6/7.4/7.3
	-CH	3.74/3.77/3.77	dd/dd/dd	6.0,9.5/6.0,9.9/6.6,9.7
Succinate (Suc)	-CH_2_	2.41/2.41/2.43	s/s/s	-/-/-
Aspartate (Asp)	-CH	2.79/2.81/2.80	dd/dd/dd	4.2,17.6/4.4,17.7/4.4,17.8
Creatine (Cr)	-CH_3_	3.01/3.01/3.02	s/s/s	-/-/-
	-CH_2_	3.91/3.91/3.91	s/s/s	-/-/-
Cho/PC/GPC*5	-CH_3_	3.19/3.20/3.19	s/s/s	-/-/-
Cho	-CH_2_	4.10/n.o./n.o.	m/n.o./n.o.	4.1,8.4/n.o./n.o.
PC	-CH_2_	4.21/n.o./n.o.	dd/n.o./n.o.	4.2,7.7/n.o./n.o.
Ethanolamine (EA)	-CH_2_	3.13/n.o./3.14	t/n.o./t	5.4/n.o./5.5
*Myo*-inositol (m-Ins)	-CH	3.26/3.28/3.27	t/t/t	9.2/9.2/9.3
	-CH	3.52/3.53/3.52	dd/t/t	3.0,10.3/10.3/10.4
	-CH	3.60/3.61/3.61	t/t/t	9.9/9.8/9.7
	-CH	4.05/4.06/4.06	s/s/s	-/-/-
*Scyllo*-inositol (s-Ins)	-CH	3.33/3.35/3.34	s/s/s	-/-/-
Taurine (Tau)	-CH_2_	3.40/3.41/3.41	t/t/t	6.9/6.8/6.7
U-Glc	-CH	5.59/n.o./n.o.	d/n.o./n.o.	5.9/n.o./n.o.
Uridine(Ud)	-CH	5.88/n.o./n.o.	d/n.o./n.o.	8.1/n.o./n.o.
	-CH	5.91/n.o./n.o.	d/n.o./n.o.	4.6/n.o./n.o.
	-CH	7.87/n.o./n.o.	d/n.o./n.o.	8.1/n.o./n.o.
Adenosine (Ade)	-CH	6.07/n.o./n.o.	d/n.o./n.o.	6.0/n.o./n.o.
	-CH	8.16/n.o./n.o.	s/n.o./n.o.	-/n.o./n.o.
	-CH	8.30/n.o./n.o.	s/n.o./n.o.	-/n.o./n.o.
Fumaric acid (Fum)	-CH	6.48/n.o./n.o.	s/n.o./n.o.	-/n.o./n.o.
Tyrosine (Tyr)	-CH	6.83/n.o./n.o.	d/n.o./n.o.	8.2/n.o./n.o.
	-CH	7.12/n.o./n.o.	d/n.o./n.o.	8.2/n.o./n.o.
Uracil (Ura)	-CH	7.47/n.o./n.o.	d/n.o./n.o.	7.68/n.o./n.o.
NAD	-CH	8.12/n.o./n.o.	s/n.o./n.o.	-/n.o./n.o.
	-CH	8.37/n.o./n.o.	s/n.o./n.o.	-/n.o./n.o.
Unassigned		7.52/n.o./n.o.	s/n.o./n.o.	-/n.o./n.o.
		7.84/n.o./n.o.	s/n.o./n.o.	-/n.o./n.o.

*1The left, centre, and right values in the lists are the results obtained from standard *J*RES, DDF*J*RES, and MAS, respectively.

*2Multiplet patterns are defined as singlet (s), doublet (d), triplet (t), quartet (q), double doublet (dd), and multiplet (m).

*3n.o. = not observable.

*4NAAG = N-Acetyl aspartate glutamate.

*5Cho/PC/GPC = Choline/Phosphocholine/sn-Glycerophosphocholine.

**Table 2:** Assignments of NMR spectra in a postmortem fish (Siamese algae eater) obtained using DDF*J*RES and standard *J*RES methods

		DDF*J*RES/*J*RES*1		
Metabolites	Groups	^1^H chemical shifts (ppm)	Multiplet patterns	*J*-couplings (Hz)
		**0.5 h postmortem**		
n-3 f.a.	-CH_3_	1.04/n.o.	t/n.o.	7.8/n.o.
All f.a. except 20:5 and 22:6	-(CH2)_n_-	1.39/1.40	s/s	-/-
All f.a. except 22:6	-CH_2_-CO	2.36/n.o.	s/n.o.	-/n.o.
Unsaturated f.a.	-N-CH_3_	5.31/5.42	s/s	-/-
Anserine (Ans)	-CH_3_	3.23/n.o.	t/n.o.	6.5/n.o.
Creatine (Cr)	-CH_3_	2.98/3.01	s/s	-/-
	-CH_2_	3.89/n.o.	s/n.o.	-/n.o.
Lactate (Lac)	-CH_3_	1.32/n.o.	d/n.o.	7.2/n.o.
	-CH	4.09/n.o.	q/n.o.	7.2/n.o.
Taurine (Tau)	-CH_2_	3.39/n.o.	t/n.o.	6.7/n.o.
		**72 h postmortem**		
All f.a. except n-3	-CH_3_	0.92/0.93	d/t	6.8/5.3
All f.a. except 20:5 and 22:6	-(CH2)_n_-	1.35/1.37	s/s	-/-
β-Hydroxybutyrate (BHB)	-CH_3_	2.18/n.o.	s/n.o.	-/n.o.
Lactate (Lac)	-CH_3_	1.38/n.o.	d/n.o.	7.3/n.o.
Acetate (Ace)	-CH_3_	1.87/1.89	s/s	-/-
Glutamate/Glutamine (Glu/Gln)	-CH_2_	2.04/n.o.	m/n.o.	7.7/n.o.
	-CH	3.70/n.o.	t/n.o.	7.3/n.o.
Pyruvate (Pyr)	-CH_3_	2.36/2.35	s/s	-/-
Creatine (Cr)	-CH_3_	2.97/2.98	s/s	-/-
	-CH_2_	3.82/n.o.	s/n.o.	-/n.o.
Betaine (Bet)	-CH_3_	3.20/3.21	s/s	-/-
Taurine (Tau)	-CH	3.38/n.o.	t/n.o.	6.7/n.o.
*Myo*-inositol (m-Ins)	-CH	3.56/n.o.	dd/n.o.	10.1&5.4/n.o.
	-CH	3.61/n.o.	dd/n.o.	9.8&5.5/n.o.
Adenosine (Ade)	-CH	8.26/n.o.	s/n.o.	-/n.o.

*1The left and right values in the lists are the results obtained from DDF*J*RES and standard *J*RES, respectively.
