# Supplementary material for: A high-resolution 2D J-resolved NMR detection technique for metabolite analyses of biological samples
Source: Sci Rep. 2015 Feb 11;5:8390. doi: 10.1038/srep08390 (PMC4323641; doi:10.1038/srep08390)
Supplement: Supplementary Information [file srep08390-s1.pdf]

Supplementary Information

A high-resolution 2D *J*-resolved NMR detection technique for metabolite analyses of biological samples

Yuqing Huang, Zhiyong Zhang, Hao Chen, Jianghua Feng, Shuhui Cai & Zhong Chen*

Department of Electronic Science, Fujian Provincial Key Laboratory of Plasma and Magnetic Resonance, State Key Laboratory of Physical Chemistry of Solid Surfaces, Xiamen University, Xiamen, Fujian 361005, China

*Correspondence and requests for materials should be addressed to Z.C. (chenz@xmu.edu.cn)

I. Theoretical expression for signals from the DDF*J*RES sequence

In the present work, the classical distant dipolar field (DDF) treatment[S1] is applied to deduce the theoretical expression of signals from the DDF*J*RES sequence. Compared to the standard 2D *J*RES technique, a fundamental requirement of the DDF*J*RES approach is that one component of the sample (e.g. solvent) is needed to generate a DDF. After the DDF modulation, the desired solute signal can be obtained. Hence, we consider a solution consisting of *I* (solvent) and *S* (solute) components, where *I* is a single spin-1/2 system and abundant for creating a DDF, and *S* is an AX spin-1/2 system that includes *J*-coupled S_k and S_l spins (coupling constant J_{kl}). Assume that ω_m is the frequency offset of the *m* ($m = I, S_k, S_l$) spins in the rotating frame in the absence of field inhomogeneity, and that $\Delta B(z)$ is the field inhomogeneity at position *z*. If the magnetization is fully modulated and varies only in one direction, the DDF will be localized and an exact theoretical expression for DDF*J*RES can be obtained. For simplification, effects of radiation damping, diffusion, relaxation, and intermolecular NOE are ignored. The function of solvent *I* is to produce a DDF for solute signals and a SS module is used before acquisition to suppress the solvent signal, therefore the theoretical expression of the solvent signal is ignored in the following. Moreover, since S_k is similar to S_l in an AX spin-1/2 system, only S_k is considered in the theoretical deduction. When the DDF*J*RES sequence is applied, the *I* spin magnetization contains a spatially modulated longitudinal component, $0.5I_z\cos\{4\gamma G_E z[\omega_I+\gamma\Delta B(z)]/R+3\gamma G\delta z\}$, which is generated after the second solvent-selective $(\pi/2)_x^I$ RF pulse. According to the classical DDF treatment, the DDF $B_d^S(z)$, originating from solvent *I* and acting on solute *S*, is given by

$$B_d^S(z)=\frac{2}{3}B_d^I(z)=-\frac{\Delta_s}{3\gamma\tau_d}\cos\{4\gamma G_E z[\omega_I+\gamma\Delta B(z)]/R+3\gamma G\delta z\}, \tag{S1}$$

where $\tau_d^I = (\gamma\mu_0 M_0^I)^{-1}$ is the dipolar demagnetizing time of I spins, in which γ is the gyromagnetic ratio for proton, μ_0 is vacuum magnetic permeability and M_0^I is the equilibrium magnetization per unit volume of I spin; $\Delta_s = [3(\hat{\boldsymbol{s}}\cdot\hat{\boldsymbol{z}})^2 - 1]/2$, in which $\hat{\boldsymbol{s}}$ is the unit vector along the CSG direction, and $\hat{\boldsymbol{z}}$ is the unit vector along the direction of static magnetic field. Since the gradient field is oriented along the z direction, i.e. $\hat{\boldsymbol{s}} = \hat{\boldsymbol{z}}$, we have $\Delta_s = 1$. G and δ are strength and duration of the first CSG, respectively; G_E is the strength of the spatial encoding gradient. The frequency sweeping rate of frequency-swept π pulses is given by $R = \Delta\nu_{ad}/\tau_{ad}$, in which $\Delta\nu_{ad}$ is the sweep width and τ_{ad} is the duration of frequency-swept π pulses.

After the modulation of the DDF shown in Eq. (S1), the observable signal of the solute S_k from the DDFJRES sequence is given by

$$M_{S_k}^{+}(t_2,t_3) = \frac{-M_0^S}{2} J_1\left(\frac{2\Delta + t_2 + t_3}{3\tau_d^I}\right) e^{-i4\gamma G_E z[\omega_I + \gamma\Delta B(z)]/R} \times \left\{ e^{i\left[\pi J_{kl} t_2 + \left(\omega_{S_k} + \gamma\Delta B(z) + \pi J_{kl}\right)t_3\right]} e^{i2\Delta\pi J_{kl}} + e^{i\left[-\pi J_{kl} t_2 + \left(\omega_{S_k} + \gamma\Delta B(z) - \pi J_{kl}\right)t_3\right]} e^{-i2\Delta\pi J_{kl}} \right\}, \tag{S2}$$

in which $J_1\left(\frac{2\Delta + t_2 + t_3}{3\tau_d^I}\right)$ is the first order Bessel function; 2Δ is delay interval for the spin echo scheme. Eq. (S2) provides quantitative expression of the 3D DDFJRES signal of solute spin S_k. The term $e^{-i4\gamma G_E z[\omega_I + \gamma\Delta B(z)]/R}$ represents the spectral information in the first evolution period t_1 (F1 dimension), $e^{\pm i\pi J_{kl} t_2}$ gives the spectral information in the second evolution period t_2 (F2 dimension), and the spectral information in the acquisition period t_3 is contained in $e^{i\left(\omega_{S_k} + \gamma\Delta B(z) \pm \pi J_{kl}\right)t_3}$ (F3 dimension). Clearly, the field inhomogeneity $\Delta B(z)$ is eliminated along the F2 dimension, but it remains in both F1 and F3 dimensions. Hence, it is unlikely to extract a high-resolution 2D J-resolved spectrum from such a 3D DDFJRES data based on direct Fourier transform. A processing for the 3D DDFJRES data can be performed to recover a 2D

J-resolved spectrum free of the influence of field inhomogeneity. The data processing method is described in the following.

II. Processing method for 3D DDF*J*RES data

From Eq. (S2), it can be noticed that the original 3D DDF*J*RES signal holds the spectral information $e^{-i4\gamma G_E z[\omega_I+\gamma\Delta B(z)]/R}$ in the first evolution period t_1, $e^{\pm i\pi J_{kl}t_2}$ in the second evolution period t_2, and $e^{i\left(\omega_{S_k}+\gamma\Delta B(z)\pm\pi J_{kl}\right)t_3}$ in the acquisition period t_3, respectively. Ω_1, Ω_2, and Ω_3 are used to represent the signal information along these three dimensions. Thus the original 3D DDF*J*RES signal can be simply expressed as

$$\left(\Omega_1,\Omega_2,\Omega_3\right)=\left(e^{-i4\gamma G_E z[\omega_I+\gamma\Delta B(z)]/R},e^{\pm i\pi J_{kl}t_2},e^{i\left(\omega_{S_k}+\gamma\Delta B(z)\pm\pi J_{kl}\right)t_3}\right). \tag{S3}$$

When a 1D Fourier transform is directly performed on the F3 dimension of the 3D DDF*J*RES signal, a batch of 1D spectra along the F3 frequency domain can be obtained, and the spectral information along the F3 dimension can be expressed as $\omega_{S_k}+\gamma\Delta B(z)\pm\pi J_{kl}$. Due to the field inhomogeneity, all these 1D spectra suffer from inhomogeneous line broadening. To recover high-resolution information from such a signal, we perform the following data processing. First, the processing for spatial encoding and decoding schemes, which arrange the train of signal echoes into a 2D matrix and then perform the Fourier transform the direct domain, is performed on F1 and F3 dimensions of the 3D DDF*J*RES signal, so *ni* 2D spectra in the F1-F3 plane can be obtained, where *ni* is the incremental number of the second evolution period t_2. Then Eq. (S3) becomes

$$\left(\Omega_1,\Omega_2,\Omega_3\right)=\left(\omega_I+\gamma\Delta B(z),e^{\pm i\pi J_{kl}t_2},\omega_{S_k}+\gamma\Delta B(z)\pm\pi J_{kl}\right). \tag{S4}$$

Inhomogeneous line broadening remains in both F1 and F3 dimensions, resulting in signal stretching along F1 and F3 dimensions. When the spectrometer reference frequency is

set to the resonant frequency of I spin, i.e. $\omega_I = 0$, all signal streaks parallel to one another and position along the center of the F1 dimension. A shearing process in the F1-F3 plane can then be performed for all 2D spectra to eliminate the inhomogeneous line broadening along the F3 dimension, expressed as

$$\begin{aligned}(\Omega_1, \Omega_2, \Omega_3') &= (\Omega_1, \Omega_2, \Omega_3 - \Omega_1) \\ &= \left(\gamma \Delta B(z), e^{\pm i\pi J_{kl} t_2}, \omega_{S_k} + \gamma \Delta B(z) \pm \pi J_{kl} - \gamma \Delta B(z)\right) \\ &= \left(\gamma \Delta B(z), e^{\pm i\pi J_{kl} t_2}, \omega_{S_k} \pm \pi J_{kl}\right).\end{aligned} \tag{S5}$$

This shearing results in signal streaks parallel to the F1 dimension in all 2D spectra of the F1-F3 plane. It can be noticed that the spectral information along the F3 dimension is free of field inhomogeneity and a projection of all these 2D spectra along the F1 dimension will give high-resolution information of chemical shifts and J couplings along the F3 dimension. Since the second evolution period t_2 forms a spin echo scheme, J couplings free of field inhomogeneity will be presented in the F2 dimension after simple 1D Fourier transform on $e^{\pm i\pi J_{kl} t_2}$. Consequently, a high-resolution 2D spectrum constructed from F2 and F3 dimensions can be obtained,

$$(\Omega_2, \Omega_3') = \left(\pm \pi J_{kl}, \omega_{S_k} \pm \pi J_{kl}\right). \tag{S6}$$

Eq. (S6) shows that the signal in the processed 2D DDF*J*RES spectrum is free of field inhomogeneity and is located at $(-\pi J_{kl},\ \omega_{S_k} - \pi J_{kl})$ and $(\pi J_{kl},\ \omega_{S_k} + \pi J_{kl})$. This 2D spectrum can be tilted by 45° to completely separate chemical shifts and J couplings information. It is obvious that spectral features in the 2D DDF*J*RES spectrum acquired in inhomogeneous fields are the same as those observed in a standard 2D J-resolved spectrum obtained in homogeneous fields. Therefore, a high-resolution 2D J-resolved spectrum can be recovered from inhomogeneous fields by the DDF*J*RES method. In practical experiments, due to intrinsic resolution defect of spatial encoding and decoding scheme[S2], the spectral resolution along the F3 dimension is lower than that along the F2 dimension.

III. Application of the DDF*J*RES method on intact shishamo smelt eggs

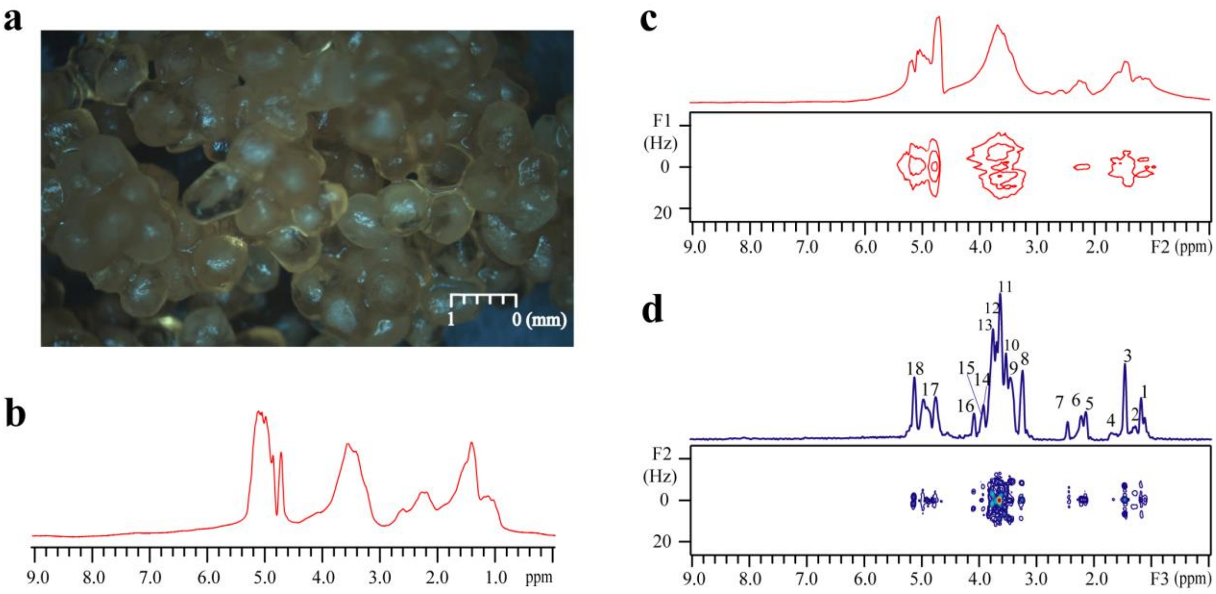

Figure S1 | Experimental results of intact shishamo smelt eggs. (a) A photo of shishamo smelt eggs taken by an electronic mircoscope; (b) 1D water-presaturated spectrum; (c) standard 2D water-presaturated *J*RES spectrum and its projection along the F2 axis; (d) 2D DDF*J*RES spectrum and its projection along the F3 axis. The peaks marked from 1 to 18 are assigned.

Figure S1 shows experimental results of intact shishamo smelt eggs. In this experiment, the sample of intact shishamo smelt eggs was fitted into a 5-mm NMR tube and then measured on a typical NMR spectrometer (500-MHz Varian NMR system with a 54-mm narrow bore). The standard 1D water-presaturated sequence, the standard 2D water-presaturated *J*RES sequence, and the DDF*J*RES sequence were applied. It is evident that the spectral resolution and spectral quality is significantly improved in the 2D DDF*J*RES spectrum (Fig. S1d) compared to the traditional 1D spectrum (Fig. S1b) and 2D *J*RES spectrum (Fig. S1c). Eighteen peaks observed in the 2D DDF*J*RES spectrum was assigned[S3]. The ^{1}H chemical shifts, multiplet patterns, and *J* coupling constants for assigned metabolites were listed in Table S1.

TABLE S1 | Assignments of intact shishamo smelt eggs in the 2D DDF*J*RES spectrum

TABLE S1 | Assignments of intact shishamo smelt eggs in the 2D DDF*J*RES spectrum

Peaks	Metabolites	Proton	^{1}H chemical shifts (ppm)	Multiplet patterns	*J* (Hz)
1	n-3 f.a.	$-CH_3$	1.09	t	7.4
2	Lactate	$-CH_3$	1.30	d	7.2
3	f.a.	$-CH_2-$	1.44	t	7.0
4	All f.a. except 22:6	$-CH_2-CH_2-COOH$	1.64	s	—
5	Unsaturated f.a.	$-CH_2-CH=CH$	2.11	s	—
6	All f.a. except 22:6	$-CH_2-COOH$	2.24	s	—
7	Glutamine	$-CH_2-$	2.43	m	4.8
8	Anserine	$-CH_2-$	3.23	t	7.5
9	Taurine	$-CH_2-$	3.43	t	6.7
10	Glycerol	$-CH_2-$	3.57	m	6.5
11	Anserine	$-CH_3$	3.65	s	—
12	Glycerol	$-CH_2-$	3.70	m	6.5
13	Phospholipid	—	3.80	m	5.6
14	Serine	$-CH_2-$	3.93	m	5.8
15	Creatine	$-CH_2-$	3.94	s	—
16	Lactate	$-CH_2-$	4.10	q	7.2
17	Residual water	H_2O	~	s	—
18	Glyceryl	$-CH_2-$	5.19	d	3.9

f.a. = fatty acid

IV. Application of the DDF*J*RES method on a semisolid sample of fruit jelly

In the experiment of fruit jelly, the sample was fitted into a 5-mm tube and measured on a typical NMR spectrometer (500-MHz Varian NMR system). Experiments were performed without field shimming and locking while the probe was well tuned to preserve high sensitivity. The standard 1D water-presaturated sequence, the standard 2D water-presaturated *J*RES sequence, and the DDF*J*RES sequence were applied for measurements. The experimental results are shown in Fig. S2. It is obvious that the spectral resolution and spectral quality is significantly improved in the 2D DDF*J*RES spectrum (Fig. S2d) compared

to the traditional 1D spectrum (Fig. S2b) and 2D *J*RES spectrum (Fig. S2c). Compared to the spectral resolution in the traditional NMR spectra, the spectral resolution in the 2D DDF*J*RES spectrum is reduced to 30 Hz along the F3 dimension and to 3.2 Hz along the F2 dimension. Observed peaks from the main chemical components (including saccharide, carrageenan, sucrose, and glucose) are well-resolved and assigned in the 2D DDF*J*RES spectrum. In addition, the *J* coupling information for these components can be obtained along the F2 dimension of the 2D DDF*J*RES spectrum.

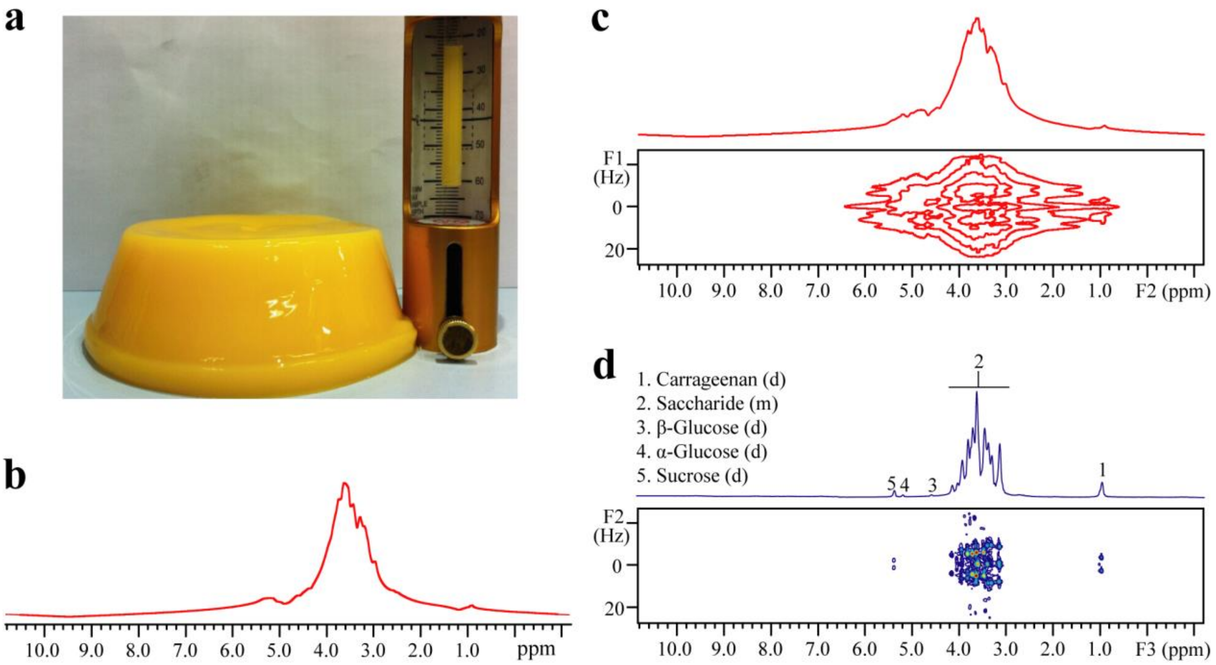

Figure S2 | Experimental results of a semisolid sample of fruit jelly. (a) A photo of the fruit jelly and the measured sample fitted into a 5-mm NMR tube; (b) 1D water-presaturated spectrum; (c) standard 2D water-presaturated *J*RES spectrum and its projection along the F2 axis; (d) 2D DDF*J*RES spectrum and its projection along the F3 axis. According to the main chemical components shown in the packaging box of the fruit jelly, peaks marked from 1 to 5 are assigned in the 2D DDF*J*RES spectrum. Multiplet patterns of *J* couplings, defined as doublet (d) and multiplet (m), are also presented in the 2D DDF*J*RES spectrum.

V. Application of the DDF*J*RES method on a viscous sample of facial cream

In this experiment, the sample of facial cream was also fitted into a 5-mm tube and measured on a typical NMR spectrometer (500-MHz Varian NMR system). Experiments were

performed without field shimming and locking while the probe was well tuned to preserve high sensitivity. The standard 1D water-presaturated sequence, the standard 2D water-presaturated *J*RES sequence, and the DDF*J*RES sequence were applied for measurements. From the experimental results shown in Fig. S3, it is clear that the spectral resolution and spectral quality is significantly improved in the 2D DDF*J*RES spectrum (Fig. S3d) compared to the traditional 1D spectrum (Fig. S3b) and 2D *J*RES spectrum (Fig. S3c). Benefiting from the spectral resolution enhancement in the 2D DDF*J*RES spectrum, five observed peaks from the main chemical components contained in the facial cream sample are assigned. And the *J* coupling information for these chemical components also can be extracted along the F2 dimension of the 2D DDF*J*RES spectrum.

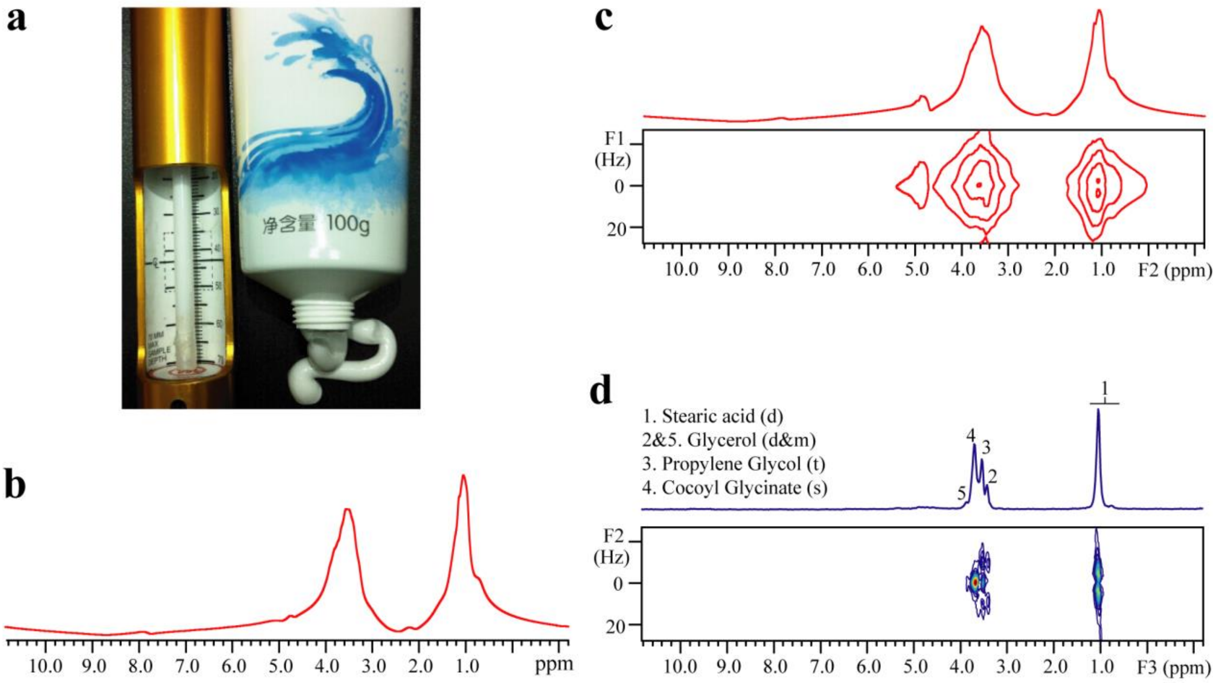

Figure S3 | Experimental results of a viscous sample of facial cream. (a) A photo of the facial cream product and the measured sample fitted into a 5-mm NMR tube; (b) 1D water-presaturated spectrum; (c) standard 2D water-presaturated *J*RES spectrum and its projection along the F2 axis; (d) 2D DDF*J*RES spectrum and its projection along the F3 axis. According to the main chemical components contained in this facial cream sample, peaks marked from 1 to 5 are assigned in the 2D DDF*J*RES spectrum. Multiplet patterns of *J* couplings, defined as singlet (s), doublet (d), triplet (t), and multiplet (m), are also presented in the 2D DDF*J*RES spectrum.

References

[S1] Jeener, J., Vlassenbroek, A. & Broekaert, P. Unified derivation of the dipolar field and relaxation terms in the bloch-redfield equations of liquid NMR. *J. Chem. Phys.* **103**, 1309-1332 (1995).

[S2] Tal, A. & Frydman, L. Single-scan multidimensional magnetic resonance. *Prog. Nucl. Magn. Reson. Spectrosc.* **57**, 241-292 (2010).

[S3] Gribbestad, I. S., Aursand, M. & Martinez, I. High-resolution 1H magnetic resonance spectroscopy of whole fish, fillets and extracts of farmed Atlantic salmon (Salmo salar) for quality assessment and compositional analyses. *Aquaculture*. **250**, 445-457 (2005).
